# A participatory study of information and presentation priorities for a digital interface for bowel self-management

**DOI:** 10.1177/20552076261491823

**Published:** 2026-09-28

**Authors:** Andrea Taylor, Gloria Iyawa, Wendy Maltinsky, Sally Henton, Dylan Lake, Sara Medina-Lombardero, Alex Casson, Michael Crichton

**Affiliations:** 1 Glasgow School of Art, School of Design, Glasgow, UK; 2 7046 University of Salford, Faculty of Science, Engineering and Environment, Salford, Greater Manchester, UK; 3 7622 University of Stirling, Psychology Division, Faculty of Natural Science, Stirling, UK; 4 3120 Heriot-Watt University, School of Engineering and Physical Sciences, Edinburgh, UK; 5 The University of Manchester, Department of Electrical and Electronic Engineering, Manchester, UK

**Keywords:** Difficulty controlling or emptying the bowel, bowel self-management, digital interface, faecal incontinence, participatory design, prototyping, self-tracking, smartphone app

## Abstract

**Background:**

Difficulty controlling or emptying the bowel is a common issue with devastating physical and mental health impacts. Digital technologies supporting bowel self-management remain limited. We are exploring a system combining an implanted sensor with a digital interface to support bowel self-management. This paper reports on the digital interface.

**Objectives:**

To identify the information most valuable to adults living with bowel problems and the best ways to present it on the digital interface, informed by lived experience; secondarily, to gather their views on the sensor and implantation methods.

**Methods:**

A participatory design approach was employed. Thirteen adults with IBD/IBS attended design sessions; five more with IBD/IBS and eight with other bowel problems attended evaluation sessions. Participants were primarily UK-based, female, and White. Design sessions explored interface priorities and lived experiences; evaluation sessions assessed usability and utility. Views on the sensor were gathered across both phases. Data were analysed using qualitative methods.

**Results:**

Five themes were identified: balancing discretion, clarity, and stigma in presentation; balancing benefit and burden; encouraging engagement without exacerbating anxiety; supporting the move from data to information to knowledge; and supporting clinical communication and confidence. Ninety-two percent of participants were positive towards sensor implantation.

**Conclusions:**

This study addresses a gap in the literature on digital interfaces for bowel self-management in combination with implantable sensors. It highlights faecal incontinence as a key patient concern, identifies priorities for information and presentation, and informs future sensor development. The work may have some limited applicability to other implantable/wearable sensors with digital interfaces.

## Introduction

Difficulty controlling or emptying the bowel is a common health issue worldwide,^1,2^ including challenges such as faecal incontinence, constipation, a lack of sensation or urge to defecate, and incomplete evacuation. For brevity, we refer to ‘difficulty controlling or emptying the bowel’ as ‘bowel problems’ in this paper. Bowel problems are associated with a range of conditions including: faecal incontinence^3^ and constipation,^4^ which may present as symptoms or be diagnosed as conditions; inflammatory bowel disease (IBD), such as Crohn’s disease and ulcerative colitis^5^; irritable bowel syndrome (IBS)^6^; neurogenic bowel dysfunction, which is the loss of normal bowel function caused by nerve problems and common in people with spinal cord injury, including cauda equina syndrome, multiple sclerosis, Parkinson’s disease, and spina bifida^7^; diabetes^8^; and low anterior resection syndrome (LARS), a complication following rectal cancer surgery.^9^ Bowel problems can have a devastating impact on an individual’s physical and mental health, including quality of life. Faecal incontinence is associated with intense embarrassment, shame and limitations in daily activities (e.g., remaining at home or near toilet facilities), and remains a widely taboo topic,^10,11^ while idiopathic constipation is highly correlated with anxiety and depression.^12^

Intestinal transit refers to the movement of digestive contents, including stool and wind, through the digestive system. Gaining a clearer understanding of this movement could help individuals develop more effective strategies for bowel self-management, which, in turn, can improve physical and mental health outcomes, including quality of life.^10,12^

Digital technology is increasingly integrated into our lives and recognised as a key enabler of global healthcare improvement.^13^ Within this broader landscape, implantable sensors (sensors placed inside the body) have the potential to revolutionise healthcare by enabling direct, real-time acquisition of critical physiological signals from internal organs and tissues.^14^ In this context, we are exploring a novel system that combines an implantable sensor with a digital interface for bowel self-management, offering possibilities beyond existing self-tracking and bowel-management tools that rely on manual data entry. The system aims to provide personalised insights into intestinal transit for improved self-management and health outcomes. While the interface was prototyped as a smartphone app, we refer to it more broadly as a digital interface to reflect a conceptual framing that moves beyond a single device or format.

The sensor is an elastomeric strain sensor, powered by a battery and connected to electronics for data transmission. It is designed to detect strain changes in the bowel when stool is passing and transmit this data to the digital interface in real-time. The sensor is intended to be fully implanted by a clinical care team. The larger project identified the sigmoid colon, the last section of the large intestine before the rectum, as the most promising location for the sensor, to allow measurements to be taken with enough warning for the patient to act. An in vitro test rig was created to support sensor development and reduce reliance on animal tissue. Preliminary testing on a bowel phantom (an artificial bowel model) demonstrated the sensor’s potential to repeatedly detect intestinal transit, with the flexibility for either internal or external placement on the large intestine. Sensor accuracy, sensitivity, and rates of false positives/negatives are not yet known and will need to be further explored in future studies using human participants. A related paper in preparation will report on the development and validation of the sensor, including the measurement and transmission electronics, aspects of which have been published.^15^

The focus of this study is the exploration of the digital interface, as all interaction occurs through it; the sensor itself has no interactive components. The primary objective was to identify the types of information most valuable to participants and the best ways to present it on the digital interface to improve bowel self-management, informed by participants’ lived experiences. The secondary objective was to gather participants’ views and preferences on the sensor and methods of implantation. Together, these objectives were intended to inform future sensor development. We note that the anticipatory aspects discussed later in this paper are hypothetical, though informed by preliminary sensor development work, and are not yet confirmed by sensor data. For clarity, in this paper, ‘bowel movement’ refers to stool and ‘wind’ refers to excessive wind.

To situate this study within the landscape of digital health technologies, we first review existing work on digital tools for bowel management and personal health tracking.

Our previous scoping review of digital technologies for bowel management identified a small but growing body of research with generally positive results on health outcomes, and highlighted implantable sensors for bowel problems as a novel area of research.^16^ Six studies met the eligibility criteria, of which two are particularly relevant here. Matsumoto et al. demonstrated improved bowel management in an older adult with severe chronic constipation using a handheld ultrasound device.^17^ Bird et al. designed a digital bowel movement tracking system for young people with autism spectrum disorder, in which care providers recorded bowel movements using an electronic form, with email notifications sent to nursing or administrative staff, e.g., if bowel movements were irregular or of poor quality. The system showed high implementation integrity and social validity.^18^ Unlike these manual-entry approaches, our system is being developed to link sensor-derived data with a digital interface, offering the possibility of anticipatory information about bowel activity. Also, these studies did not actively involve people with lived experience in the design of the technology, whereas our study used a participatory design approach. While not included in the six studies, Vega et al. found that mobile apps for faecal incontinence symptom tracking are scarce, and emphasised that tracking is crucial for assessing severity, as increased frequency of episodes is associated with decreased quality of life.^19^ They recommended that future app development include symptom tracking specific to faecal incontinence to potentially improve health outcomes and involve both practitioners and patients, which, as noted, our study does.

In addition, recent work by O’Connor et al. (2025) identified research priorities for faecal incontinence (FI) through a James Lind Alliance priority-setting partnership.^11^ Among these priorities was a call for “new and novel approaches to managing FI,” including technologies such as stem cells and bioelectronics, highlighting the need for innovation in this area.

Beyond bowel-specific literature, a growing body of work in personal informatics focuses on data collection for personal health, including chronic conditions.^20^ Ayobi et al. found that self-tracking practices in multiple sclerosis help individuals regain control by integrating self-care practices with tools like fitness trackers and meditation apps.^21^ They emphasise that self-tracking should support overall wellbeing, not just primary disease indicators. Later, Ayobi et al. developed a customisable self-tracking app allowing users to create pictorial trackers based on personal needs, highlighting the importance of supporting individual needs and creative engagement with health data.^22^ Luo et al. reported that existing food trackers often fail to meet the diverse needs of people with dietary problems and can be improved through customisability.^23^ Co-design sessions with dietitians identified five tracking categories: food, reflection, activity, symptoms, and physical state. Bussone et al. reported that current technologies do not meet the tracking needs of people living with HIV.^24^ The authors identified that important aspects for tracking tools include the ability to handle large, fluctuating data, support for gaining health insights, data ownership, security, privacy, and clinician connection. These studies collectively highlight that existing tracking tools may not fully support the diverse and individualistic needs of people with chronic conditions, and that flexible, customisable tools which extend beyond primary disease indicators are needed to support improved self-management. Our study extends this prior work by introducing new design considerations around how sensor-derived information might be presented, interpreted, and integrated into daily life.

We also explored the commercial landscape by searching app stores for publicly available tools. While apps such as Poop Tracker,^25^ Poop Log,^26^ plop,^27^ and FI Stool Diary Instrument^28^ exist, they are primarily designed for journaling and do not provide mechanisms for sensing movements in the bowel or providing anticipatory insights.

## Methods

This study adopted a qualitative, participatory design approach. Participatory design actively involves the people being served through design in the process, recognising that they are the experts of their own experience and that their contributions are essential to finding solutions to problems.^29^ Within this approach, we used prototyping as a key method to facilitate collaborative exploration of participants’ experiences and ideas. This study was reported in accordance with the Standards for Reporting Qualitative Research (SRQR) guideline^30^ to enhance methodological transparency and rigour (details in Supplemental material 1). Authors 1–5, the main contributors to this study, have experience in qualitative research and digital health. Aware that our personal attributes and assumptions could shape interpretation, we used iterative participant feedback during the participatory process as our primary reflexive mechanism to check and refine our interpretations.

This study comprised two phases: design sessions and evaluation sessions. These sessions were conducted between May 2022 and May 2023, during which all data were collected. A series of five design sessions focused on identifying the types of information participants considered most valuable and the best ways to present it on the digital interface for improved bowel self-management, informed by their lived experiences of bowel problems. The evaluation sessions gathered participants’ early reactions and reflections on the content, perceived usefulness, and overall utility of the digital interface. Participants were divided into two groups for the evaluation sessions, with the rationale for this division explained later. Group 1 participated in individual interviews, and Group 2 in a focus group. Both phases also gathered participants’ views on the sensor and methods of implantation.

Each session lasted two hours and was delivered online via Microsoft Teams, following expert advice that online participation is easier for those requiring quick access to the toilet. Digital whiteboards (Google Jamboards) were used to facilitate the sessions, e.g., to share prototypes of the digital interface and capture participants’ contributions on sticky notes.

This study was approved by The Glasgow School of Art and University of Stirling research ethics committees. Verbal informed consent was obtained electronically from all participants (see Consent to Participate under Statements and Declarations for details). All data were stored securely, and participant contact details were destroyed after the results summary was distributed. Pseudonyms are used to help maintain participant anonymity; participants chose their own pseudonyms, except for two, for whom Author 1 assigned the names Irene and Walter.

### Participants

Participants were selected using purposive sampling. We aimed to recruit 12 participants for the design sessions, in line with guidance that sample adequacy in purposive qualitative research is based on achieving thematic saturation.^31,32^ For the evaluation sessions, we aimed to recruit five participants in Group 1, which is considered sufficient for an initial round of usability testing,^33^ and eight participants in Group 2 as part of the same saturation-based qualitative approach. By the end of the study, we judged that thematic saturation had been reached with respect to the study’s primary objective of identifying information and presentation priorities.^31,32^ This judgement was based on the analysis process described under Data Analysis, in which contributions from later design sessions continued to fit within existing information needs, and evaluation sessions did not identify any new information or presentation priorities. Inclusion criteria were: aged 18+ years; a history of difficulty controlling or emptying the bowel; and owning a digital device, such as a smartphone or laptop. Recruitment was conducted through advertisements shared via the social networks of bowel-health charities. Each participant was paid for their time and expertise.

Twenty-six participants were recruited for this study. Thirteen took part in the design sessions (Table 1), with an average of nine participants per session (range: 6–11). Although this study focused on bowel problems more broadly, all design session participants were individuals living with IBD and/or IBS. This was unplanned and emerged organically from the recruitment process. The evaluation sessions were therefore conducted with two participant groups: Group 1 and Group 2. Group 1 was planned from the outset and included individuals with the same bowel problems as those in the design sessions, ensuring the prototype was reviewed by people with similar lived experiences. Group 2 was added later to explore whether the content of the prototype digital interface was also valuable to people with different bowel problems. Thirteen people were recruited to the evaluation sessions (Table 2): five for Group 1 and eight for Group 2. None took part in both the design and evaluation sessions.

**Table 1.** Participants who took part in the design sessions, and which of the five sessions they participated in, indicated by “X”.

​	Session 1	Session 2	Session 3	Session 4	Session 5
Blackcat	​	X	X	X	X
Cosmonaut	​	​	X	​	​
Dan	X	X	X	X	X
Irene	X	​	​	X	X
KP	​	X	X	​	X
Lucy	​	X	​	X	X
Maggie	X	​	X	​	​
Meg	​	X	X	​	X
Moo	X	X	X	X	X
PAT	X	X	X	X	X
Sausage	X	X	X	X	X
Shorthouse	​	X	X	X	X
Star	​	X	​	X	X
**Total**	6	10	10	9	11

**Table 2.** Participants who took part in the evaluation sessions, and which of the two groups they participated in.

Group 1	Group 2
Anna	Alf
Baxter	Bonnie
Lamppost	Clyde
Martha	Gordon
Sorcha	Liduc
​	RaRa
​	Scarlett
​	Walter

Demographic information was collected using an online questionnaire (Table 3), which, due to the sensitive nature of the research topic, was anonymous to maximise confidentiality and foster a safe environment for sharing experiences.

**Table 3.** Participant demographics. DS=Design sessions; ES1=Evaluation Sessions–Group 1; ES2=Evaluation Sessions–Group 2.

Variable	Category	DS n	ES1 n	ES2 n
Gender	Female	11	5	4
	Male	2	​	4
Age	16-24	1	​	​
	25-34	1	2	​
	35-44	4	1	1
	45-54	3	1	1
	55-64	3	​	5
	65+	1	1	1
Ethnicity	Asian, Asian Scottish, Asian British	​	​	1
	Mixed or Multiple ethnic group	2	​	​
	White	11	5	7
Employment status	Paid full-time	9	3	2
	Paid part-time	​	​	1
	Retired	1	1	3
	Student	1	​	1
	Unemployed	​	1	1
	Unpaid work	2	​	2
Country/s of residence	Canada	​	​	1
	Dubai	1	​	​
	United Kingdom	13	5	7
Main bowel problem/s	Cuada equina syndrome	​	​	1
	Diabetes	​	1	1
	IBD	11	5	​
	IBS	5	2	1
	LARS	​	​	1
	Multiple sclerosis	​	1	2
	Nerve/muscle damage	​	1	2
	Neurogenic bowel disorder	​	​	1
	Parkinson’s disease	​	1	2
	Spinal cord injury	​	1	3
Onset of bowel problem/s	Up to 1 year ago	​	​	1
	1-5 years ago	5	2	5
	5-10 years ago	2	​	​
	10+ years ago	6	3	1
	Not sure	​	​	1

### Design Sessions

#### Identifying Information and Presentation Priorities

Session 1 explored participants’ lived experiences of bowel problems to prime the identification of information and presentation priorities for the digital interface. This ‘day in the life’ activity^29^ invited participants to create a story of a typical day using a timeline template divided into ‘morning’, ‘afternoon’, and ‘night’. The template included three key questions: What experiences do you have controlling or emptying your bowel during these periods? How do you monitor and/or manage your experiences? How effective are these approaches? The template was sent in advance to allow time for preparation.

Also conducted during Session 1, the user needs statements activity^34^ built on the personal experiences shared in the ‘day in the life’ activity. It encouraged participants to revisit specific situations they had described and consider what information would be most valuable in those moments. The activity used a template with three open-ended sentence prompts to guide reflection on informational needs: ‘When I …’ (the situation in which the challenge occurs), ‘I need/want…’ (the information that would be most valuable in that situation), and ‘So that…’ (the desired outcome).

Session 2 focused on brainstorming ideas to address each identified need individually, using a scenario-based approach. Each scenario was structured in three parts: the first and third parts (‘When I…’ and ‘So that…’) were pre-populated with results from Session 1 to remind participants of the situations and desired outcomes. The middle section (‘I need…’) was titled with the corresponding need and left blank. Participants were invited to generate ideas for this section to explore, in more depth, the types of information that would be most valuable and the best ways to present them on the digital interface.

Sessions 3–5 focused on exploring participants’ earlier contributions in greater depth through iterative discussion. A progressively refined static (non-interactive) prototype was prepared between sessions to support these discussions, serving as a way of ‘building to think’— a phrase from co-design literature that refers to moving beyond the capture of experience and insight, to embody participant voices and bring a degree of tangibility to ideas.^35^ Session 4 also explored the visual appearance of the information, e.g., the colour palette, and how visual elements might influence engagement.

Following Session 5, the static prototype was refined. In parallel, after Session 3, an interactive (semi-functional) version of the prototype was created, as part of the wider development work to read data transmitted by the sensor, and for use in the evaluation sessions. For clarity, the interactive prototype did not incorporate real sensor data. Development of the interactive prototype followed a rapid, incremental approach using an agile software development method where the content and presentation of information were continuously added and refined, as far as practicable. In healthcare, rapid prototyping has been described as producing “transitional objects” that support a change from a current behaviour to a new one, enabling participants to imagine and engage with new behavioural possibilities.^35^ While the prototype was not designed for near-term implementation, its semi-functional nature allowed it to act in this way within this study.

#### Gathering Views on the Sensor and Implantation

Sessions 2–5 also included gathering participants’ views and preferences on the sensor and methods of implantation. Two main methods were proposed: an endoscopic procedure to attach the sensor within the bowel and a laparoscopic procedure to attach the sensor outside the bowel. Key questions included: Would you potentially be happy to have the sensor implanted in your body? What method of attachment would you be most comfortable with and why? This topic was revisited across sessions to ensure that all participants had the opportunity to share their views, and to capture evolving perspectives as the sensor design developed and more detailed information became available.

### Evaluation Sessions

**Group 1** sessions were conducted individually and included a standard user-centred design (UCD) usability testing method and think-aloud protocol.^36^ The session had two parts. Part 1 involved a usability test using the interactive prototype. A researcher ran the prototype in a mobile app simulator on their desktop computer, shared their screen, and enabled remote control so the participant could interact with it. Participants engaged in basic tasks (e.g., recording a bowel movement), with a primary focus on the usability of the interface and how easily the information could be understood. Best practices were followed, such as emphasising that it was the interface that was being tested, not the participants. Part 2 used the static prototype to walk through content not yet incorporated into the interactive version. Participants were invited to reflect on the usability and overall utility of the information presented.

**Group 2** participated in a focus group session. The session used the static prototype to walk through the content of the digital interface, largely due to the ability to demonstrate workflows more easily, i.e., display multiple screens at the same time. As with Group 1, participants were asked to reflect on the usability and utility of the information using the same key questions.

Across both groups, participants shared their views and preferences regarding the sensor and methods of implantation, using the same key questions asked during the design sessions.

#### Data Analysis

All sessions were audio-recorded and transcribed using automated software, with transcripts checked against the recordings before coding.

**Lived Experiences**: Inductive reflexive thematic analysis^37^ was used for the ‘day in the life’ activity. Data familiarisation with transcripts was conducted independently by Authors 3 and 4. Codes were developed by Author 4 to evoke the main data features. Initial themes captured patterns from these data features. Reflective discussions between the two authors led to iterative evolution and refinement of themes with reference to potential bias.

**Information and Presentation Priorities**: Author 1 used Affinity diagramming^36^ for the user needs statements activity and ordered the resulting information needs based on the extent to which they were represented across participant contributions. The information needs were reviewed by Authors 2–4, checked with participants in Session 2, revisited in subsequent sessions, and iteratively refined. After Session 2, Author 1 mapped and clustered subsequent contributions from the design sessions against these evolving needs, translated them into design decisions for the digital interface in collaboration with Author 5, and had them reviewed by participants. Author 1 also used affinity diagramming for the evaluation sessions. The Group 1 and 2 results were analysed separately, then combined, synthesised, and translated into refinements for the digital interface, with no new information or presentation priorities identified beyond those established in the design sessions. This approach was taken because the key topics, questions, and prototypes were fundamentally the same across groups, and careful comparison of the results revealed no meaningful group-level differences. These refinements were reviewed by Authors 2 and 5.

Author 1 subsequently developed overarching themes by clustering and synthesising results across the design and evaluation sessions, which were reviewed by all authors. We report these overarching themes in the Results because they offer broader conceptual value, while the specific design decisions derived from them are included in Supplemental material 2.

**Sensor**: Data were descriptively organised by Author 1, with responses clustered under the key questions.

These three analytic strands are reported separately in the Results section as Lived Experiences, Information and Presentation Priorities, and Views on the Sensor and Implantation.

## Results

### Lived Experiences

Four themes that were persistent patterns in participants’ lived experiences of bowel problems were identified:

**1. Challenges of the unknown—a mental toll.** This theme focuses on the emotional impact of unpredictability, both of the condition and of other people’s responses. It includes challenges with bowel volatility (such as frustration when patterns or triggers are unclear and fear and worry about what might happen), and challenges with cultural awareness (including embarrassment, shame, stigma, and pressures to eat or drink in social situations).
**2. Optimising quality of life.** This theme captures participants’ goal-oriented adjustments to reduce bowel symptoms and improve quality of life. It includes controlling oral intake (such as choosing not to eat in order to go out, or eating plain foods to minimise overnight symptoms), and careful scheduling (such as avoiding travel when symptoms are worse or rising early to allow time for a bowel movement).
**3. When the going gets tough.** This theme reflects participants’ use of workarounds and distractions to manage symptom flares. It includes mental refocusing (such as playing phone games for distraction and using positive thinking), and minimising repercussions (such as being prepared with emergency kits (incontinence pads, wipes, etc.) and knowing the location of toilets).
**4. Impossible choices.** This theme reflects participants’ need to make the most of imperfect choices. It includes the perception of the need to struggle on or admit defeat (such as a day being shaped by symptoms like pain), engaging in activities regardless of bowel problems (such as finding a bush for a bowel movement at an outdoor gathering), and engaging with minimal risk (such as cooking without eating the food or attending social gatherings without eating/drinking).

### Information and Presentation Priorities

This section begins by outlining four information needs identified in the design sessions, presented in descending order based on the extent to which they were represented across participant contributions. Following this, five overarching themes are presented on the types of information most valuable to participants, and the best ways to present it on the digital interface to support improved bowel self-management. Here, presentation refers not just to visual aspects, but more broadly to how, when, and in what format information is delivered. While each theme reflects a distinct area of focus, they are closely interconnected. To support the thematic findings, selected screens from the prototype digital interface are included to illustrate how participants’ preferences around information and presentation were explored and reflected during this study.

#### Information Needs

Faecal incontinence was identified by nearly all participants as the most significant concern, reflected in the following information needs:

**1. To have more certainty that a bowel movement and/or wind could occur.** Participants mostly needed more certainty regarding a bowel movement, with enough notice to reach a toilet. Sorcha shared, “I’m pretty much housebound… I just don’t take part in stuff now because I basically ruin it when something goes wrong.” Participants also needed more certainty about the best time to attempt a bowel movement, due to constipation or lack of urge or sensation. Gordon explained they had adapted by going “on the clock… to sort of think about it every few hours.”
**2. To have more certainty about what is going on in the bowel.** Participants wanted to overcome bowel unpredictability by identifying and understanding patterns and triggers for movements in the bowel, e.g., “if you could see, you could avoid certain foods in certain situations” (RaRa). As Dan shared, “overall, I would like to be in control of where things are, because for me, the most annoying thing is … the uncertainty, anything that could potentially give me some sort of information would be ideal.”
**3. To have more certainty about whether it is a bowel movement and/or wind.** Some participants needed more certainty about whether what they were experiencing was stool and/or wind, with enough notice to act. Liduc shared, “I don’t have sensation, I don’t know if it is wind or the toilet… makes it very difficult for family, so informing if there is bulk, that I need to get to a toilet fast would improve the quality of my life.” Sausage wanted to know when wind was building so they could move physically to avoid “loads and loads of pain.”
**4. To have more certainty whether there is more stool in the bowel.** Some participants needed more certainty about whether the bowel was fully emptied. Gordon shared, “often I have [an accident] coming off the toilet where I think I’m finished, but my nerves don’t know that I am.” Some also valued knowing when a bowel movement hadn’t occurred for a specific period, to “do something” (PAT) and avoid constipation or obstruction. As RaRa noted, “that can be helpful medically because I get concerned about like a bowel obstruction.”

#### Overarching Themes

##### Balancing Discretion, Clarity, and Stigma in Presentation

A recurring theme was the importance of discretion in presenting information about bowel activity, particularly in public, while also ensuring sufficient clarity when timely action might be required, as “this is important stuff” (Bonnie). Participants proposed designs that are recognisable to those using the digital interface but not to others, including the use of abbreviations such as “BM” (bowel movement) and “W” (wind) in notifications and icons (Figure 1), a stylised colon as the app icon, described as “[not] overtly obvious, yet not terribly vague” (Blackcat), and the name *Sensolon* (a blend of ‘sensor’ and ‘colon’), which balanced recognisability and discretion. Participants also preferred colour schemes that avoided associations with stool, such as green and blue, e.g., “red, orange, yellow and brown are just a no-no really” (PAT). Several suggested a smartwatch version, e.g., “it’s smaller … the chance of somebody else seeing is quite small” (Dan), and proposed notification settings that could override usual sound or vibration preferences, e.g., “forcing sound only for this specific app … [is] a clear trigger that I need to check my phone” (Dan). A few cautioned that overemphasis on discretion could reinforce stigma, e.g., not talking about bowel health is “why so many people end up with bowel cancer, etc., and having problems” (RaRa).

**Figure 1.**
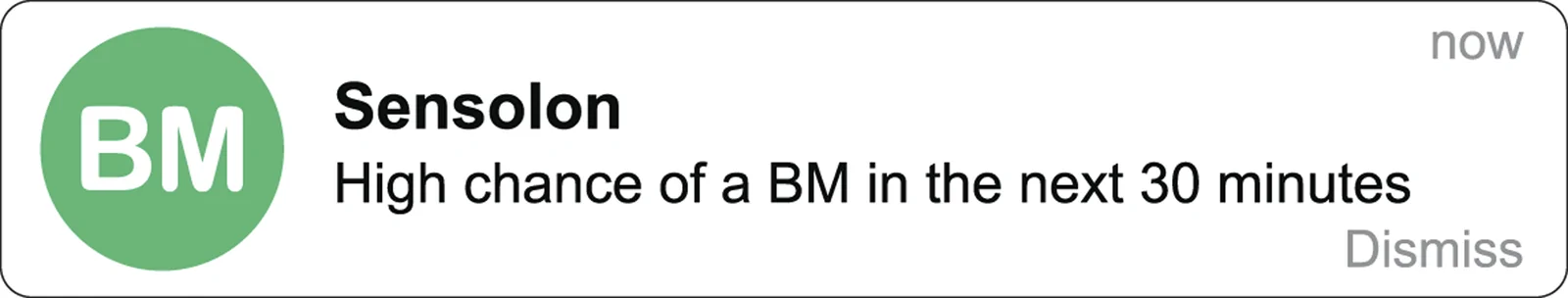
Mock-up of a bowel movement (“BM”) notification designed to be clear to the person using the app but discreet to others nearby.

##### Balancing Benefit and Burden

Participants recognised that for the app to become reliable and useful, it needed to learn from their experiences, meaning that each bowel movement/episode of wind, and associated symptoms should ideally be recorded. Automatic timestamping of predicted movements was welcomed. Participants also valued the option to record further details. As Dan put it, “the more you feed it, the better it’s going to do for you.” Participants identified the following optional measures: accident, blood, evacuation, mucous, pain, recovery time, texture, time spent on the toilet, and volume (Figure 2). They agreed on predefined values (e.g., ‘No’, ‘Near’, ‘Yes’ for accident) and supported existing tools like the Bristol Stool Chart, while acknowledging its limitations, e.g., “[it’s] not perfect, but a good place to start” (KP). Beyond bowel-specific data, participants valued tracking aspects of daily living such as activity, food, medication, mood, and sleep, e.g., “[medication] has a big effect on my bowel … I think most people with bowel trouble really struggle to keep track of which medication is working best” (Gordon). However, participants were mindful of the burden of data entry, “to a point where it becomes daunting” (Dan), especially with frequent bowel movements, and suggested the flexibility for individuals to track only the measures most useful to them, potentially based on clinician advice (Figure 3). Dan reflected on personal trade-offs: “am I willing to put in all that data, no, then maybe I’m fine with only receiving partial data that’s helpful.” To support tracking while minimising burden, participants welcomed a reminder notification that opened a prepopulated tracking form with editable sensor data: date, time, and type of episode (bowel movement and/or wind). Some also saw the app as a temporary tool to help identify patterns rather than a long-term commitment. To reduce effort, participants proposed tracking templates and integration with existing apps such as Apple Health or MyFitnessPal, e.g., “that could all be automated” (Walter).

**Figure 2.**
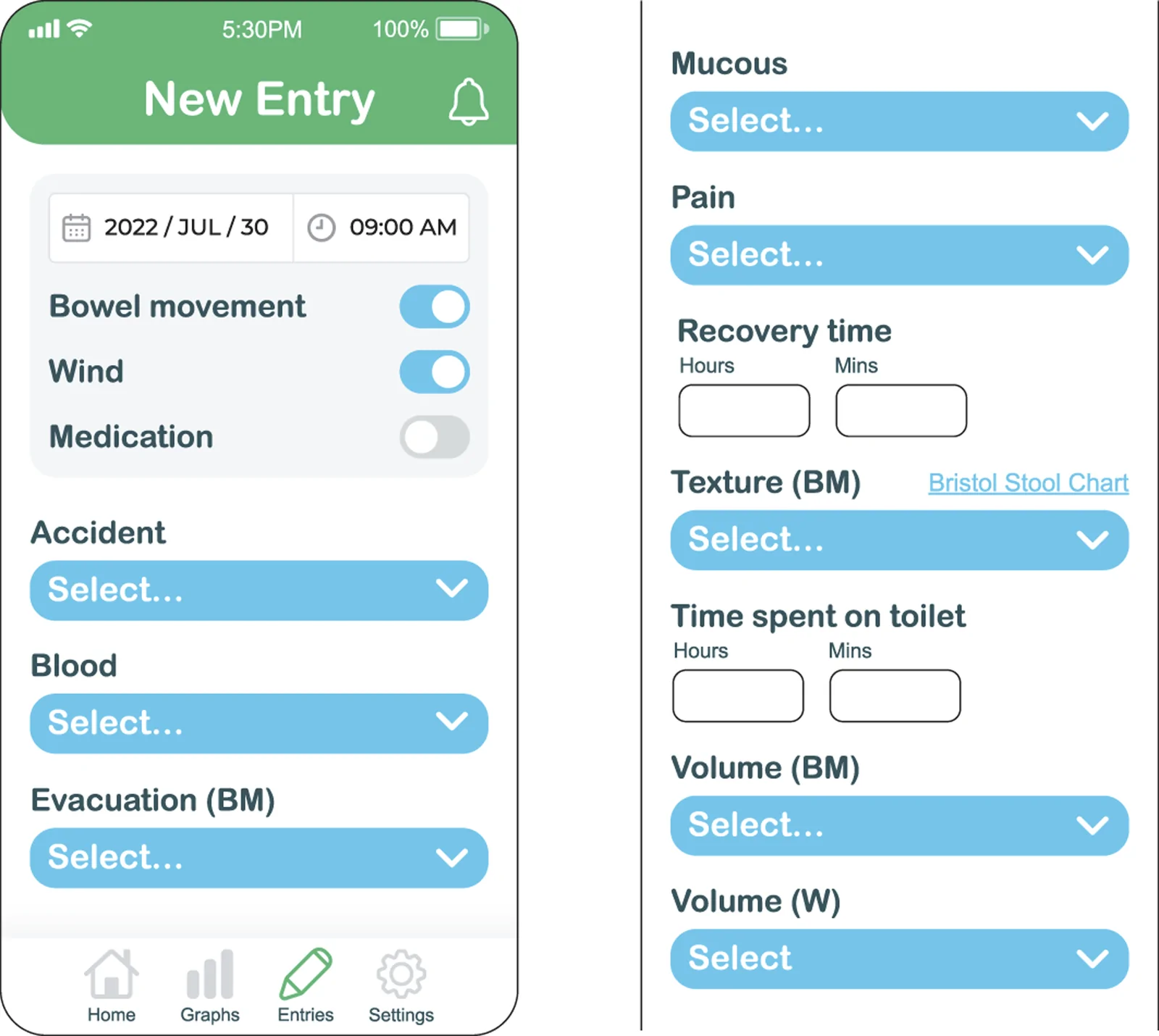
Mock-up of a form to record a bowel movement, and/or wind, and/or taking medication (all of which can happen at the same time), illustrating the optional measures relevant to bowel movement and wind.

**Figure 3.**
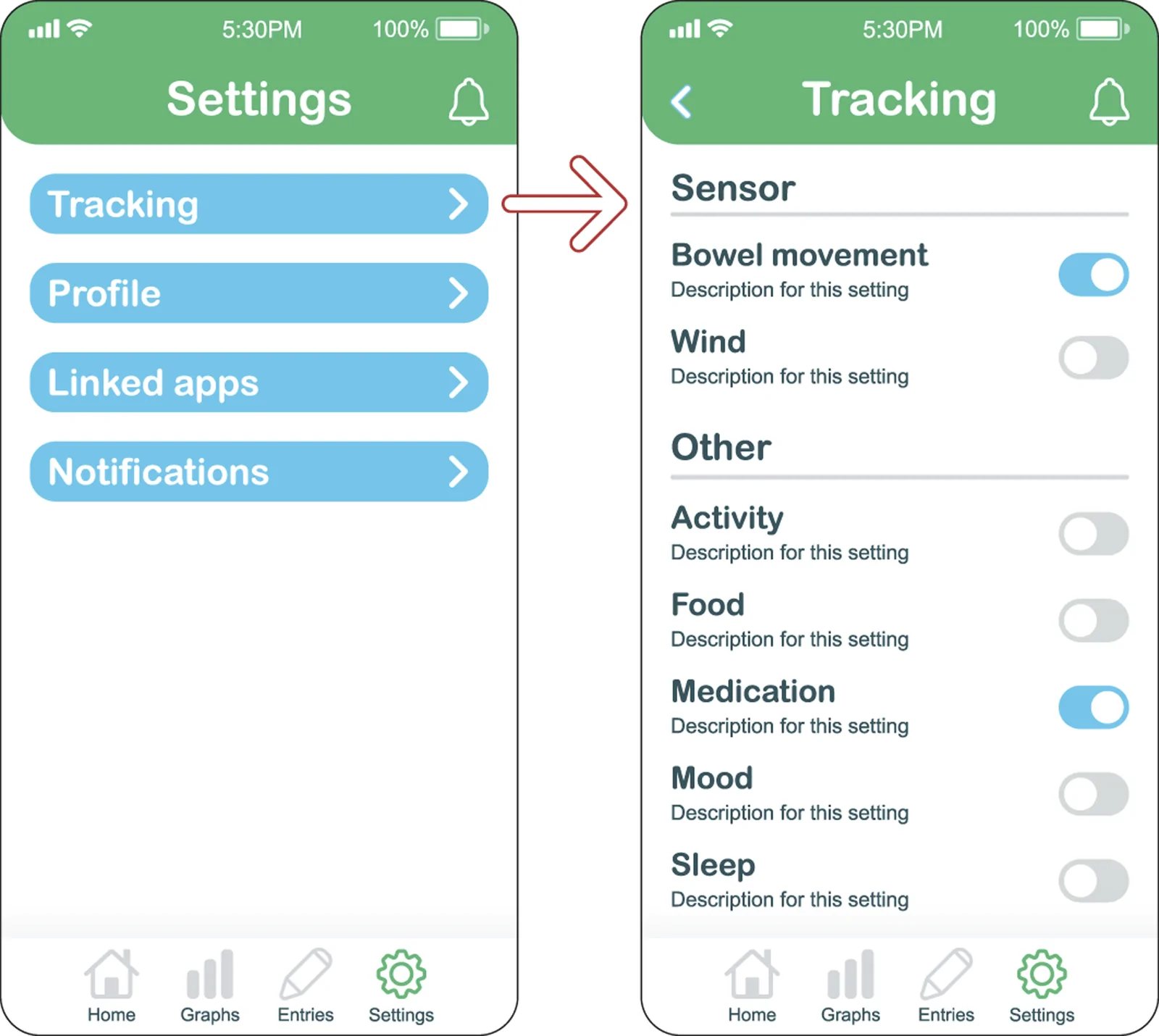
Mock-up of settings showing how individuals can customise which aspects of bowel activity and daily living are tracked.

##### Encouraging Engagement Without Exacerbating Anxiety

Participants agreed on the value of push notifications to alert of an imminent bowel movement or to prompt that now would be an optimal time to attempt one. Participants proposed receiving these notifications 30 minutes in advance, and all agreed that this timeframe would allow adequate time to reach a toilet or otherwise prepare, e.g., “to take… some pain relief because you have to wait about half an hour or whatever for this codeine to work whilst you’re in agony” (Moo). However, an important consideration raised was that some individuals can have a high number of bowel movements, e.g., “on a bad day, 25” (Sorcha). They may also occur multiple times in a short period, e.g., “six times in the space of half an hour” (Sausage). Given this, there was concern that frequent notifications might increase anxiety, e.g., “you could have your phone going off, going off, going off, and you’re quietly having a nervous breakdown in the corner” (Bonnie). Consequently, participants proposed the ability to customise the notification threshold based on the likelihood of a bowel movement occurring, using a simple three-point scale: ‘Low’, ‘Medium’, ‘High’ probability. Some participants also wanted notifications for specific scenarios, such as when no bowel movement is detected for a set period or when wind is building. In both cases, they emphasised the importance of customisable thresholds and the ability to opt out, as these features may not be relevant to everyone. Views on when to send reminder notifications varied, given the high frequency of bowel movements among some participants. Suggestions included customisable timings or a single end-of-day notification.

##### Supporting the Move from Data to Information to Knowledge

Participants wanted to move beyond collecting data toward transforming it into knowledge, including anticipatory information, to support self-management. They particularly wanted a graphical display showing the probability of a bowel movement on the current or following day, e.g., “some sort of chart that shows, with these probabilities, this is going to happen” (Dan). Blackcat wanted the ability to set a more granular timeframe, “say, for the next two hours or whatever I’m going to be out and about, is anything going to happen.” Participants also valued information that could help identify patterns and triggers. This included wanting to understand why things changed (“if I’ve gone to the loo fewer times… what is it based on?”, Dan), prevent accidents (“the more information I have, hopefully the fewer accidents I have”, Sausage), make practical adjustments (“if you could see [a pattern], you could avoid certain foods in certain situations”, RaRa), and reduce anxiety when planning activities (“knowing these sorts of things… could reduce some of that pre-anxiety… I always want the seat on the end”, Moo). Visualising data over time was seen as essential, with Martha commenting that mapping different types of information together helped “to see the correlation” (Figure 4). However, making sense of the data was seen as a challenge. Dan cautioned, “you can’t just have everything on a graph,” and suggested preset graphing options “to say what goes with what”, so that only relevant types of information are shown together. Gordon similarly emphasised the need for a clear display aligned with a personal “target state”.

**Figure 4.**
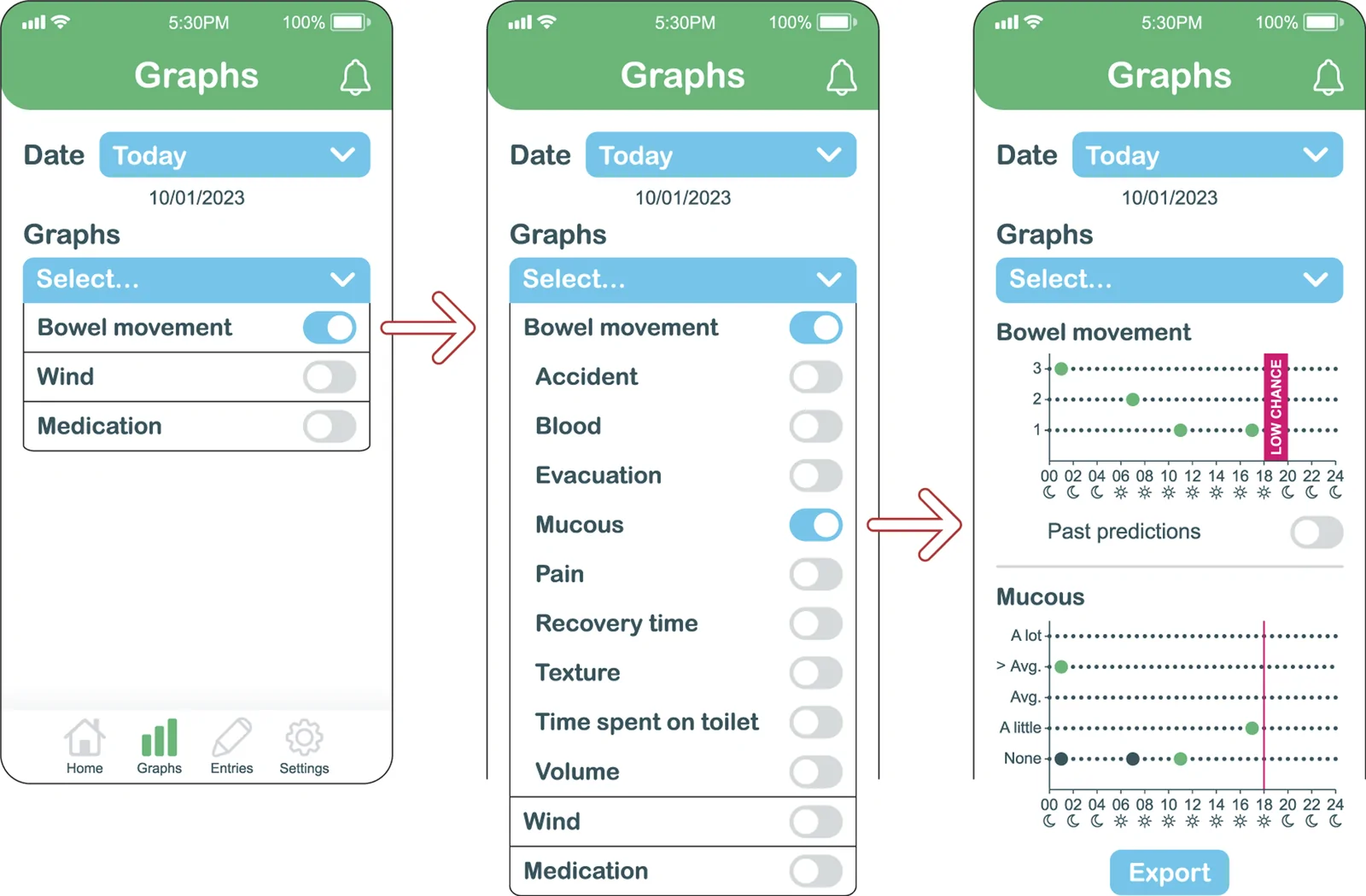
Mock-up of a graphical display showing “Bowel movement” (quantity) mapped against “Mucous” for “Today”.

##### Supporting Clinical Communication and Confidence

Participants viewed the app as a tool to support communication with clinicians by making experiences easier to convey, including the ability to export data in a visual format (“when I have to send [data] to my GP… I just send in the graph, and you can read it really easy”, PAT), making visible what might otherwise be minimised with “I’m fine” in consultations (“proof that you haven’t been fine”, Blackcat), and supporting recall of details that may be hard to remember (“has there been mucus… has there been blood”, Baxter). Cosmonaut also emphasised the importance of different clinicians, “doctors, GPs, gastros and even dietitians”, having access to the same data. The app was also seen to overcome limitations of paper diaries, such as poor adherence and inconsistent data entry. Participants also highlighted the value of recording incorrect predictions to build confidence in the system, e.g., “to give that negative that there was no bowel or wind movement… a negative validation as well as a positive one” (Walter). Dan suggested that preset graphing options should be developed with clinician input, “to say what goes with what,” so that the app supports tracking measures relevant to clinical care.

### Views on the Sensor and Implantation

Participants were positive about the sensor. Liduc remarked, “it would make such a quality-of-life change to have more control… to be able to socialise”, and almost everyone (92%) reported they would potentially be happy to have it implanted. Regarding implantation methods, seven (27%) preferred endoscopy, citing it as a routine procedure that could be combined with an annual colonoscopy to reduce discomfort, e.g., “to kill two birds with one stone would be cool” (KP). Four (15%) preferred laparoscopy, mainly due to prior discomfort/pain with colonoscopy, while six (23%) expressed no strong preference, e.g., “either way, I think the potential benefits would outweigh concerns about having it done” (Baxter). Seven (27%) were open to implantation but would decide based on the risks, and two (8%) would not consider it due to invasiveness, e.g., “I’ve had more [than enough] operations to last a lifetime” (Lucy). Other concerns included the sensor coming loose and causing “further strictures” (PAT) or being “another sensation gurgling inside me” (RaRa).

## Discussion

In this paper, we present a participatory design study whose primary contribution is the identification of information and presentation priorities for a digital interface intended to link with an implantable bowel sensor. This novel context introduces design considerations not addressed in prior work, particularly around how sensor-derived information might be presented, interpreted, and integrated into daily life. It is important to note that the overarching themes reflect patient perspectives, and future clinician involvement will guide the next stage of iterative development (e.g., visualisations and thresholds). As additional context, most participants were open to sensor implantation, with preferences shaped by familiarity and perceived invasiveness, though concerns about risks and comfort were raised.

**Balancing discretion, clarity, and stigma in presentation** extends prior self-tracking work on socially stigmatised conditions, where users prioritised covert, low-visibility ways of recording and storing information to avoid unintended disclosure.^24^ In our context, participants similarly wanted bowel information to be discreet in public, but not so subtle that it undermined clarity when timely action is required. A few also cautioned that an overemphasis on discretion could itself reinforce stigma, highlighting that discretion is related to but distinct from traditional privacy concerns.

**Balancing benefit and burden** extends prior work by identifying a preliminary set of symptom-tracking measures that, to our knowledge, is novel, including ‘accident’ to track faecal incontinence. This responds to Vega et al.’s (2021) recommendation for symptom tracking specific to faecal incontinence and extends the measures used in prior work, e.g., Bird et al. (2022), to reflect patient priorities. Further, while prior work shows that self-tracking often becomes short-lived due to burden, our results highlight a distinct rationale: participants viewed short-term use as purposeful—a way to identify bowel patterns after which they would stop using the system.

**Encouraging engagement without exacerbating anxiety** extends prior work (e.g., Epstein et al., 2020) by highlighting how anxiety shaped by the unpredictability of bowel symptoms can be further increased when bowel activity triggers high-frequency notifications. As Dan commented, while encouraging engagement is important, you “don’t want to put more pressure on people that are embarrassed and suffering”, worrying that not recording something could lead to “an unfortunate event when I go out today.” In response, participants discussed ways to modulate engagement, e.g., adjusting when and whether notifications are sent, to minimise increased anxiety while maintaining value.

**Supporting the move from data to information to knowledge** extends prior work by highlighting the value of anticipatory information for bowel self-management. For example, while Bird et al. (2022) relied on care providers manually recording bowel movements that triggered retrospective alerts for potential constipation or obstruction, participants in this study wanted sensor-derived and participant-entered data to be combined to provide anticipatory notifications (e.g., of a bowel movement). This also contrasts with Matsumoto et al. (2020), where patients were prompted to attempt bowel movements each morning regardless of bodily cues, which, as Cosmonaut noted, can often result in “a haemorrhoid or I bleed.”

**Supporting clinical communication and confidence** extends prior work by highlighting that participants felt objective, sensor-derived data and graphs could help them communicate their experiences more clearly and confidently, particularly when details are hard to recall or easy to minimise. It is important to note that this theme reflects patient perceptions of what they would find valuable, with some imagining of clinician needs through their own lens, such as the usefulness of access to data and graphs. This highlights the importance of engaging with clinicians who might be on the receiving end of patient-generated reports.

Across the themes, the results relate to personal informatics concepts of tracking burden, data fatigue, and non-use. These concepts appeared in several ways, including challenges linked to high-frequency bowel movements, high notification frequency, and the sensitive, stigmatised nature of bowel problems. Additionally, personal informatics research highlights privacy and ethical concerns around tracked data as important concerns.^20^ While the detailed privacy, security, and consent processes will need to be developed in future collaborative work, we anticipate using a privacy-by-design approach that includes secure and encrypted handling of sensor data, minimisation of stored data, patient-controlled access for clinicians, and clear mechanisms for patients to give and withdraw consent.

### Hypotheses: Implications for Other Technologies

Bowel problems were reported by participants with a range of health conditions (Table 3). The results of this study may therefore have some limited applicability to the design of technologies for other conditions that include bowel management (e.g., tools for Parkinson’s disease) or where people may prefer monitoring and management activities to be more discreet (e.g., tools for type 1 diabetes^38^). There may also be some limited relevance to other technologies where implantable sensors could provide discreet monitoring, potentially contributing to broader discussions on how people interact with devices in their body. However, given the predominantly IBS/IBD sample and the prototype stage of the research, the results of this study should be considered preliminary.

### Contributions

This study contributes to digital health in the following ways:

• **Addressing a gap in the literature**: Our previous scoping review (reported separately) identified a relatively limited body of literature on digital technologies for bowel management, with implantable sensors highlighted as an unexplored area of research.^16^ This study begins to address this gap by exploring how a digital interface might be designed to work with an implanted sensor. In doing so, it adds preliminary work to an emerging research area and aligns with the research call for “new and novel approaches to managing FI”.^11^
• **Extending participatory design to sensor–digital interface combinations for implantable technologies**: This study applies participatory design to a prototype system that integrates an implantable sensor with a digital interface. While participatory design is well established in digital health, we are not aware of its use in the development of sensor–digital interface combinations for implantable technologies. This study, therefore, offers an early example of using participatory design to shape interface design in the context of an implantable system.
• **Highlighting patient-defined priorities**: This study identified faecal incontinence as the primary concern among participants, with significant implications for their quality of life. Despite its prominence in lived experience, faecal incontinence remains underrepresented in the design and development of digital technologies. By foregrounding this priority through participatory design, this study contributes preliminary insights into the types of information and presentation approaches that participants consider most valuable for supporting bowel self-management.
• **Informing future sensor development**: A related paper (under preparation) will report on the development and validation of the sensor. This study contributes by gathering participants’ views and preferences on implantation methods, providing a preliminary foundation for guiding future sensor development in ways that are more aligned with patient needs. While both papers form part of a larger project, this study focuses on the digital interface, recognising it as a critical component of the overall system. Separating the papers allows for a more detailed exploration of the priorities identified through participatory design.

### Limitations

#### Participant Sample

A key limitation of the participant sample is the imbalance in gender, ethnicity, and geography: 76.9% female (20/26), 88.5% White (23/26), and 96.2% UK-based (25/26, including one participant with dual residence in the UK and Dubai). Given this demographic profile, the results may not be generalisable to individuals residing outside the UK and may be less applicable to men and non-White individuals. The gender imbalance may reflect the target population, as faecal incontinence and constipation are more common among women than men.^2,3^

A further limitation is that the design session participants and Group 1 evaluation participants were individuals living with IBD/IBS. These participants largely talked about the symptoms they experienced, mostly faecal incontinence, constipation, and wind. The Group 2 evaluation participants experienced a wider range of bowel problems, but they still focused on the same core symptoms, with faecal incontinence a strong concern across both the design and evaluation sessions. We therefore tentatively suggest that the five themes may be relevant beyond the participant sample in this study.

In addition, recruitment through social networks of bowel health charities may have inadvertently biased the participant sample toward individuals who are more digitally engaged and health literate. This may have meant that participants were more comfortable with digital health technologies and better able to describe the types of information and presentation they would find most useful for supporting bowel self-management, which helped us meet our main study objective. However, the results may not fully reflect the views of individuals with lower digital access or lower digital or health literacy.

#### Clinician Involvement

While the project received valuable clinical advice, particularly from a steering group of healthcare experts, including gastroenterology and bowel control nursing, clinicians did not participate as research participants. Due to resource constraints (seed funding), we prioritised individuals living with bowel problems (experts by experience). This means the results reflect patient perspectives only.

#### Digital Interface

A smartphone app was proposed as the digital interface at the outset of this study, and this format was used throughout the participatory design process. The results suggest that an app is acceptable, desirable, and mostly practical from participants’ perspectives, which aligns with prior studies reporting good usability and patient acceptability of mobile apps for self-management in IBS and faecal incontinence.^39,40^ However, we acknowledge that beginning with a smartphone-app format will have shaped participant ideation, particularly by encouraging a focus on mobile-style interactions (e.g., notifications, icons) and offering fewer opportunities to explore alternative devices/formats that a digital interface might take (e.g., a smartwatch-based format).

## Conclusion

Bowel problems are a prevalent health issue worldwide that can have a profound emotional and practical impact on an individual’s daily life. Faecal incontinence can be particularly devastating. Participants described challenges including unpredictability, embarrassment and stigma, and the need to make difficult trade-offs. Their strategies included restricting their activities and, therefore, their quality of life, and navigating social norms and pressures. Our sensor-based system was well received by participants, with particular value placed on notifications of imminent bowel movements and building understanding of patterns and triggers for bowel symptoms.

Our planned next step is to engage with a larger and broader group of clinicians, including gastroenterologists, incontinence nurses, colorectal surgeons, urogynaecologists, urologists, physiotherapists, and psychologists interested in pelvic floor disorders. This work will include gathering their views on the clinical contexts in which the sensor-based system may be applied, as well as its acceptability, benefits, risks, barriers, and facilitators to uptake. It will explore the information clinicians consider most valuable to track, including the frequency and duration of data collection needed for patterns to emerge. It will also explore how the system would integrate into current clinical practice. These results will be compared with the present study to identify areas of alignment and divergence, informing the further development of a system that integrates both patient and clinician priorities within current clinical practice. These insights will also inform the design of future studies, including the potential expansion of recruitment to a broader range of participants.

## Supplemental material

Supplemental material - A participatory study of information and presentation priorities for a digital interface for bowel self-management Supplemental material for A participatory study of information and presentation priorities for a digital interface for bowel self-management by Andrea Taylor, Gloria Iyawa, Wendy Maltinsky, Sally Henton, Dylan Lake, Sara Medina-Lombardero, Alex Casson, Michael Crichton in Digital Health.

Supplemental material - A participatory study of information and presentation priorities for a digital interface for bowel self-management Supplemental material for A participatory study of information and presentation priorities for a digital interface for bowel self-management by Andrea Taylor, Gloria Iyawa, Wendy Maltinsky, Sally Henton, Dylan Lake, Sara Medina-Lombardero, Alex Casson, Michael Crichton in Digital Health.

## Data Availability

The datasets generated during this study are not publicly available due to their unstructured nature and dispersion across multiple sources, and because preparing them for public dissemination would require substantial resources. However, we have included a descriptive metadata document describing aspects of our research.*
