# Supplementary material for: A participatory study of information and presentation priorities for a digital interface for bowel self-management
Source: Digit Health. 2026 Sep 28;12:20552076261491823. doi: 10.1177/20552076261491823 (PMC13769949; doi:10.1177/20552076261491823)
Supplement: Supplementary file 2 [file sj-pdf-2-dhj-10.1177_20552076261491823.pdf]

Design Decisions

Needs: To have more certainty that a bowel movement and/or wind could occur (#1); To have more certainty what is going on in the bowel (#2); To have more certainty about whether it is a bowel movement and/or wind (#3); To have more certainty whether there is more stool in the bowel (#4).

Needs	Design decisions	
#1	Alert of a bowel movement, or prompt of an optimal time to attempt a bowel movement.	• Push notification, sent with 30 minutes notice.<br>• Option to set a threshold for receiving notifications, based on the probability of the prediction being correct.
#1	Graphical displays of predicted future movements in the bowel.	• Graphs for 'today' and 'tomorrow', indicating the probability of the predictions being correct.
#2, #3, #4	Tracking bowel movements and/or wind.	• Form to record an episode: date/time; bowel movement and/or wind, which determines the measures that are displayed; values for the measures. Full list of measures: accident, blood, evacuation, mucous, pain, recovery time, texture, time spent on toilet, and volume. Bristol Stool Chart for texture, with option to customize an alternative value/s.<br>• Editable repository of past entries.
#2	Tracking aspects of daily living.	• Option to track activity, food, medication, mood, and sleep.<br>• Option to manually input data or share data between apps.<br>• Form to record medications: names, dosages, and start/stop dates.
#2	Graphical displays of data to communicate bowel patterns and triggers.	• Graphs, with date picker and preset timeframes.<br>• Option to select (graph) multiple items that are being recorded/tracked.<br>• Option to display past predictions (to assess accuracy).<br>• Option to export graphs.
#2	Prompt to record a movement in the bowel.	• Reminder push notification with buttons to: open the form (above), pre-populated with data (editable) from the sensor—date, time, episode (bowel movement and/or wind); or record that the prediction was incorrect.<br>• Option to set a timeframe for the reminder.<br>• Option to schedule a single/daily reminder, which either links direct to the relevant form (if a single notification applies), or to a list of notifications (if multiple notifications apply), each linking to the relevant form.
#3	Alert of a build-up of wind.	• Push notification, indicating the probability of the prediction being correct.<br>• Option to not track wind.
#4	Alert of no movement in the bowel data.	• Push notification—optional.<br>• Option to set a timeframe for the notification.
	For all notifications:	• Option to select a more discreet wording.<br>• Option to override the main phone settings for sound and vibrate, and turn sound/vibrate on for notifications.
